# Retraction Note: Cognitive function assessed by Mini-mental state examination and risk of all-cause mortality: a community-based prospective cohort study

**DOI:** 10.1186/s12877-025-06951-0

**Published:** 2026-01-16

**Authors:** Yongkang Su, Jing Dong, Jin Sun, Yan Zhang, Shouyuan Ma, Man Li, Anhang Zhang, Bokai Cheng, Shuang Cai, Qiligeer Bao, Shuxia Wang, Ping Zhu

**Affiliations:** 1 Medical School of Chinese PLA, 28 Fuxing Road, Haidian District, Beijing, 100853 China; 2 Department of Geriatrics, the Second Medical Centre, Chinese PLA General Hospital, 28 Fuxing Road, Haidian District, Beijing, 100853 China; 3 Department of Cadre Clinic, the First Medical Centre, Chinese PLA General Hospital, 28 Fuxing Road, Haidian District, Beijing, 100853 China; 4 Department of Cardiology, the Second Medical Centre, Chinese PLA General Hospital, 28 Fuxing Road, Haidian District, Beijing, 100853 China

**Retraction Note: BMC Geriatrics (2021) 21:524**

10.1186/s12877-021-02471-9

The Editors have retracted this article.

After publication, concerns were raised that the Mini-Mental State Examination (MMSE) instrument was used without proper permissions. The authors were contacted, but they were unable to provide a satisfactory explanation or documentation for securing permissions.

On behalf of all authors, Yongkang Su stated that the authors disagree with the retraction. 

